# Bipolar disorder integrative staging: incorporating biomarkers into progression across stages (boarding-pass study)

**DOI:** 10.1192/j.eurpsy.2026.10721

**Published:** 2026-07-31

**Authors:** N. Girone, L. Cremaschi, F. Martella, B. Benatti, M. Cocchi, A. Caporali, S. Saluzzi, C. Cavallotto, I. Rosa, A. Di Giorgio, C. D’Addario, M. Pettorruso, F. de Pasquale, G. Martinotti, B. Dell’Osso

**Affiliations:** 1 Department of Mental Health, Department of Biomedical and Clinical Sciences, University of Milan, L. Sacco University Hospital, Milan; 2 Department of Bioscience and Technology for Food, Agriculture and Environment; 3 Faculty of Veterinary Medicine, University of Teramo, Teramo; 4 School of Advanced Studies, Center for Neuroscience, University of Camerino, Camerino; 5 Department of Mental Health and Addictions, ASST Papa Giovanni XXIII, Bergamo; 6 Department of Neuroscience, Imaging and Clinical Sciences, University G. D’Annunzio Chieti-Pescara , Pescara; 7 Department of Mental Health and Addictions, University of Milan, L. Sacco University Hospital; 8 ”Aldo Ravelli” Center for Neurotechnology and Brain Therapeutics, University of Milan, Milan, Italy; 9 Bipolar Disorders Clinic, Stanford University, California, United States

## Abstract

**Introduction:**

Bipolar disorder (BD) is a lifelong, recurrent condition with growing evidence supporting a neuroprogressive course, entailing the need to adopt staging models to guide stage-specific interventions^1^. Although different approaches have been proposed so far, their application remains limited and largerly based on clinical features. Recent advances in biological markers, neuroimaging, and artificial intelligence may enhance the precision of staging models, thus enabling more personalized treatment strategies^2-3^.

**Objectives:**

The study aims to: (1) longitudinally assess clinical stage progression in BD over an 18-month follow-up using the Kupka & Hillegers model; (2) investigate the contribution of epigenetic regulation, inflammatory markers, oral microbiota profiles, and structural and functional MRI connectomics to stage transitions; and (3) develop and validate supervised ML algorithms capable of integrating clinical, biological, and neuroimaging variables to provide individualized, data-driven predictions of stage progression.

**Methods:**

The BOARDING-PASS is a multicenter, prospective, observational study aimed at advancing current knowledge of BD progression.The study enrolled 120 subjects (age 18-70 years), classified according to the Kupka & Hillegers’ staging model, and recruited from three secondary-level psychiatric services in Italy. The primary outcome is the longitudinal assessment of clinical stage progression over a 18-month period, with assessment conducted at baseline (T0), T1 (6 months), T2 (12 months), and T3 (18 months after baseline). At each time point, socio-demographic and clinical variables were collected, as well as clinical stages were assigned. Additionally, at T0, T2, and T3, structural and functional MRI scans and peripheral blood and unstimulated saliva samples were collected. Finally, all multimodal data were integrated within a supervised ML algorithm, with the goal of developing a refined, data-driven staging model for BD (* Figure 1*).

**Results:**

As of September 2025, all 125 participants have been enrolled, with >50% having completed 6-month follow-up and 25% reaching 12 months. Dropout rate remains <10%. Preliminary internal analyses on partial datasets suggest promising accuracy (>70%) in predicting stage transitions with supervised ML models.

**Image 1:**
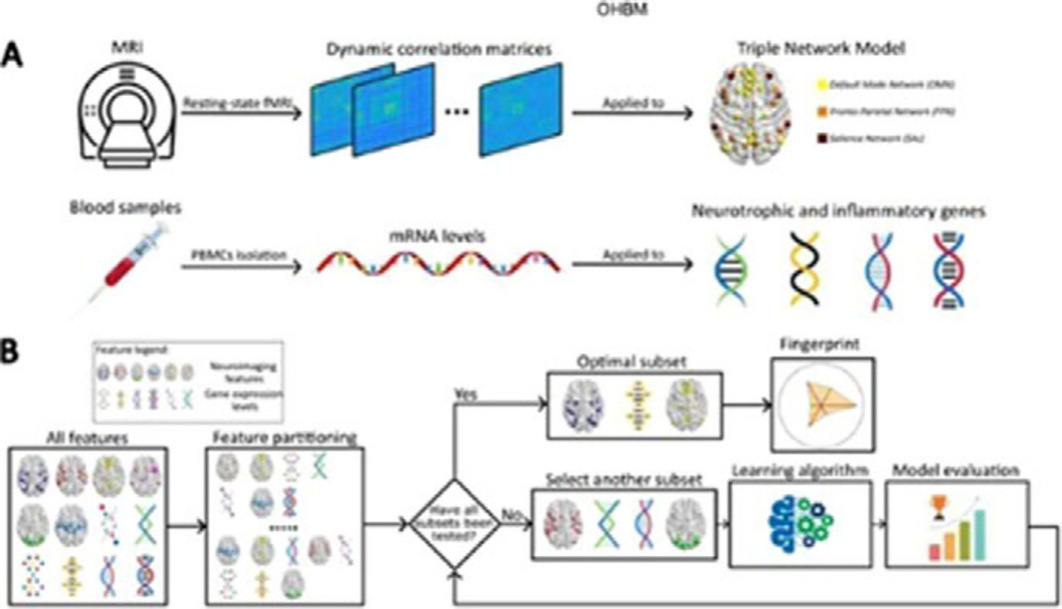
Image 1: Long description.

**Conclusions:**

The BOARDING PASS project aligns with the growing need for a standardized, biologically informed staging framework that integrates clinical, inflammatory, epigenetic, and neuroimaging profiles. **Incorporating these** dimensions may significantly enhance the characterization of BD patients by identifying specific dysfunctional trajectories and overcoming the traditional, diagnosis-centered approaches, thus leading to more targeted and effective treatment strategies.

The study is funded by the European Union - Next Generation EU - NRRP M6C2 - Investment 2.1 - PNRR-MAD-2022-12376693.

**Disclosure of Interest:**

N. Girone: None Declared, L. Cremaschi: None Declared, F. Martella: None Declared, B. Benatti Speakers bureau of: Angelini, Lundbeck, Janssen, Rovi, M. Cocchi: None Declared, A. Caporali: None Declared, S. Saluzzi: None Declared, C. Cavallotto: None Declared, I. Rosa: None Declared, A. Di Giorgio: None Declared, C. D’Addario: None Declared, M. Pettorruso Grant / Research support from: Angelini, Janssen-Cilag, and Lundbeck, Speakers bureau of: Angelini, Janssen-Cilag, and Lundbeck, F. de Pasquale: None Declared, G. Martinotti Grant / Research support from: Angelini, Doc Generici, Janssen-Cilag, Lundbeck, Otsuka, Pfizer, Servier, Recordati, Rovi, Viatris, Speakers bureau of: Angelini, Doc Generici, Janssen-Cilag, Lundbeck, Otsuka, Pfizer, Servier, Recordati, Rovi, Viatris, B. Dell’Osso Grant / Research support from: Rovi, Angelini, and Lundbeck, Speakers bureau of: Angelini, Janssen, Otzuka, and Lundbeck, Viatris and Bromatech

## Long descriptions

### Image 1: Long description

Panel A is divided into two parallel horizontal tracks. The top track begins with an M R I scanner icon labeled Resting-state f M R I, leading to Dynamic correlation matrices, which are then applied to a Triple Network Model of the brain. This model includes the Default Mode Network (D M N) in yellow, Fronto-Parietal Network (F P N) in orange, and Salience Network (S N) in dark red. The bottom track begins with a syringe icon for Blood samples, leading to P B M C s isolation and m R N A levels, which are applied to Neurotrophic and inflammatory genes represented by four D N A double helix icons.

Panel B outlines the computational workflow. It starts with a Feature legend identifying neuroimaging features and gene expression levels. A box labeled All features contains multiple brain maps and D N A icons, which flows into Feature partitioning. A decision diamond asks Have all subsets been tested? If Yes, it leads to an Optimal subset and a Fingerprint diagram. If No, it leads to Select another subset, followed by a Learning algorithm represented by a brain and gear icon, and finally Model evaluation shown as a bar graph with a trophy. An arrow loops from Model evaluation back to the decision diamond.

Navigate back to Image 1:.

